# The Jena Audiovisual Stimuli of Morphed Emotional Pseudospeech (JAVMEPS): A database for emotional auditory-only, visual-only, and congruent and incongruent audiovisual voice and dynamic face stimuli with varying voice intensities

**DOI:** 10.3758/s13428-023-02249-4

**Published:** 2023-10-11

**Authors:** Celina I. von Eiff, Julian Kauk, Stefan R. Schweinberger

**Affiliations:** 1 Department for General Psychology and Cognitive Neuroscience, Institute of Psychology, Friedrich Schiller University Jena, Am Steiger 3, 07743 Jena, Germany; 2 Voice Research Unit, Institute of Psychology, Friedrich Schiller University Jena, Leutragraben 1, 07743 Jena, Germany; 3 DFG SPP 2392 Visual Communication (ViCom), Frankfurt am Main, Germany; 4 Jena University Hospital, 07747 Jena, Germany

**Keywords:** Emotion, Audiovisual integration, Voice morphing, Stimulus database, Adaptive testing, Emotion induction, Cochlear implant

## Abstract

We describe JAVMEPS, an audiovisual (AV) database for emotional voice and dynamic face stimuli, with voices varying in emotional intensity. JAVMEPS includes 2256 stimulus files comprising (A) recordings of 12 speakers, speaking four bisyllabic pseudowords with six naturalistic induced basic emotions plus neutral, in auditory-only, visual-only, and congruent AV conditions. It furthermore comprises (B) caricatures (140%), original voices (100%), and anti-caricatures (60%) for happy, fearful, angry, sad, disgusted, and surprised voices for eight speakers and two pseudowords. Crucially, JAVMEPS contains (C) precisely time-synchronized congruent and incongruent AV (and corresponding auditory-only) stimuli with two emotions (anger, surprise), (C1) with original intensity (ten speakers, four pseudowords), (C2) and with graded AV congruence (implemented via five voice morph levels, from caricatures to anti-caricatures; eight speakers, two pseudowords). We collected classification data for Stimulus Set A from 22 normal-hearing listeners and four cochlear implant users, for two pseudowords, in auditory-only, visual-only, and AV conditions. Normal-hearing individuals showed good classification performance (*M*_corrAV_ = .59 to .92), with classification rates in the auditory-only condition ≥ .38 correct (surprise: .67, anger: .51). Despite compromised vocal emotion perception, CI users performed above chance levels of .14 for auditory-only stimuli, with best rates for surprise (.31) and anger (.30). We anticipate JAVMEPS to become a useful open resource for researchers into auditory emotion perception, especially when adaptive testing or calibration of task difficulty is desirable. With its time-synchronized congruent and incongruent stimuli, JAVMEPS can also contribute to filling a gap in research regarding dynamic audiovisual integration of emotion perception via behavioral or neurophysiological recordings.

**Supplementary Information:**

The online version contains supplementary material available at 10.3758/s13428-023-02249-4.

## Introduction

Faces and voices carry a large range of information about a person, including identity, gender, age, or emotional state (for a review, see Young et al., 2020). Especially understanding affective signals is fundamental for people, as emotions can vary dynamically and from moment to moment, and can be seen as basic for human interaction (Zajonc, 1980). It seems crucial to utilize valid and reliable stimulus sets which meet various requirements when investigating emotion recognition. Despite this, a recent survey of face stimuli used in psychological research between 2000 and 2020 highlighted a continuing preponderance of researchers to use static stimuli, with no trend for an increase over time in the proportion of studies that use dynamic (vs. static) faces (Dawel et al., 2022). We here aim to provide a stimulus set comprising dynamic facial and vocal emotional expressions and, thus, a significant resource to face and voice research. In the sections below, we discuss a number of desiderata that guided our development of the present JAVMEPS stimulus set as an open resource to promote investigations into emotion perception.

### The need for precisely time-synchronized, dynamic AV stimuli

Emotions are inherently multimodal (Klasen et al., 2012; Robins et al., 2009; Young et al., 2020). In most everyday situations, they are conveyed simultaneously through face and voice, and other communication channels like body gestures. The automatic integration of cross-modal emotional signals is underlined by findings that humans cannot inhibit the processing of emotional information of a task-irrelevant modality (Föcker et al., 2011; Takagi et al., 2015; Vroomen et al., 2001). Moreover, contrastive aftereffects on vocal emotion perception caused by perceptual adaptation can be elicited crossmodally, manifesting in a bias towards perceiving features of a stimulus that contrast with the adapted stimulus quality (Pye & Bestelmeyer, 2015). For instance, neutral voices are perceived as happier not only after adaptation to angry voices but also after adaptation to angry faces (Skuk & Schweinberger, 2013). The perception of emotional states based on only unimodal stimuli can even be recalibrated when adding (e.g., slightly discrepant) information from another modality (Baart & Vroomen, 2018; Watson & de Gelder, 2020). Neuroimaging studies found relatively early integrative responses to emotional audiovisual (AV) stimuli (Gao et al., 2019; Hagan et al., 2009; Hagan et al., 2013; Jessen & Kotz, 2011). A significant impact of visual information on early auditory processing is also suggested by the demonstration from event-related potential (ERP) studies of an auditory N1 amplitude suppression for dynamic face–voice pairs when comparing AV to an auditory-only condition (Kokinous et al., 2017). The authors of the same study also found that emotional dynamic AV cues lead to significantly reduced latencies, compared to the auditory-only condition, which also emphasizes the crucial role that integration of AV stimuli plays in emotion perception.

Importantly, whereas most currently available AV stimulus sets for emotion perception research contain only static faces (Beaupré & Hess, 2005; Ebner et al., 2010; Egger et al., 2011; Ekman, 1976; Erwin et al., 1992; Kanade et al., 2000; Langner et al., 2010; Lundqvist et al., 1998; Matsumoto & Ekman, 1988; Mazurski & Bond, 1993; Tottenham et al., 2009; Tracy et al., 2009; Wang & Markham, 1999), facial emotional expressions encode the emotion message in complex facial action patterns. Facial movement facilitates affective processing (Ambadar et al., 2005; Bassili, 1979; Biele & Grabowska, 2006; Bould et al., 2008; Cunningham & Wallraven, 2009; Krumhuber & Kappas, 2005; Krumhuber et al., 2013; Nelson & Russell, 2014; Pollick et al., 2003; Sato & Yoshikawa, 2004; Wehrle et al., 2000) and elicits differential and enhanced patterns of neural activation, compared with static expressions (Pitcher et al., 2011; Recio et al., 2011; Trautmann et al., 2009). Notably, cross-modal perception is disrupted when AV stimuli are presented in temporal incongruency. It is therefore crucial for AV integration that facial movement and vocal signals are presented in sufficient temporal synchronicity. Research with systematic experimental asynchronicities studied this temporal window of integration for speech perception via the McGurk effect; results suggest a small and asymmetric window of integration between the approximately 50-ms auditory-lead and the 200-ms auditory-lag (Munhall et al., 1996; van Wassenhove et al., 2007). For AV integration in speaker identity recognition, this window of integration appears to be slightly larger (approx. 100-ms auditory-lead to 300-ms auditory-lag) (Robertson & Schweinberger, 2010; Schweinberger & Robertson, 2017). While similar studies into emotion perception are currently lacking, the dynamic nature of emotions suggests that the relevant integration window may be more similar to the one seen in speech perception, thus making good time synchronization a priority for any stimuli that investigate AV integration in emotion perception.

### The need for stimuli which provide a controlled manipulation of emotion intensity

One of the most salient facets of an emotion is its intensity (Sonnemans & Frijda, 1994), making intensity an important aspect in theories of emotion (Diener et al., 1985; Frijda, 1988; Plutchik, 1980; Reisenzein, 1994; Schlosberg, 1954). However, surprisingly, an explicit manipulation of intensity is only given in very few AV stimulus sets (Bänziger et al., 2012; Kaulard et al., 2012; Livingstone & Russo, 2018). More (compared to less) intense vocal and facial expressions are better recognizable (Hess et al., 1997; Juslin & Laukka, 2001). Thus, using stimuli with a controlled manipulation of intensity in studies not only allows the systematic investigation of intensity itself but also creates the possibility of adjustment to different target groups in research. For example, (sub)clinical populations, such as individuals with schizotypy or autism spectrum traits, might benefit from more intense stimuli (Blain et al., 2017). A powerful tool for controlled manipulation of intensity of (e.g., emotional) voices is voice morphing technology using the STRAIGHT algorithm (Kawahara et al., 2013; Kawahara & Skuk, 2019). Voice morphing allows precise systematic control and even parametric variation of acoustic cues conveying social signals in the voice, while preserving the naturalistic sound of vocal stimuli. Correspondingly, voice morphing is increasingly used to provide new insights into how emotions in voices are processed (Bestelmeyer et al., 2010; Skuk & Schweinberger, 2013; von Eiff et al., 2022a, b). Compared to more traditional methods to calibrate auditory difficulty, voice morphing also has distinct advantages because it avoids unnecessary degradation in terms of distortion or noise in the auditory signal. Utilizing voice morphing technology is also specifically beneficial for AV stimuli because it allows the manipulation of the intensity of vocal expressions while keeping the intensity of facial expressions constant. This, for example, enables researchers to systematically investigate the specific impact of voices on AV integration. Moreover, voice morphing offers the option to produce vocal caricatures, which are modulations of vocalizations beyond the veridical emotion. Caricatured emotions, compared to original emotional voices and anti-caricatures, are perceived as higher in emotion intensity and arousal, and are recognized faster (Whiting et al., 2020).

### The need for stimuli devoid of semantic content

Using natural speech stimuli – i.e., words or sentences in an existing language – in emotion perception research appears reasonable, yet is debatable. First, research across the world done with natural speech in different languages is difficult to interrelate. Second, semantic meaning of words can influence emotion perception in a face or a voice (e.g., Gendron et al., 2012). Pseudowords – non-words composed of a combination of phonemes that conform to a language’s phonotactic rules – are one way of addressing these issues (Keuleers & Brysbaert, 2010). Pseudowords do not contain semantic meaning and thus allow for speech prosody to become the central feature of emotion processing (Wendt et al., 2003; Wendt & Scheich, 2002). We considered that pronunciation of pseudowords is usually based on pronunciation rules of a specific language (i.e., German, in case of JAVMEPS), but also that languages of one language family (e.g., the Germanic languages as a branch of the Indo-European language family which comprises English and German) should allow subsets of broadly similar combinations of phonemes. In that sense, research across the world which makes use of the same pseudoword stimuli can be more comparable than research which makes use of “normal” speech, and can potentially facilitate more general conclusions – even when cross-language validation of pseudoword stimulus sets ultimately seems desirable to control for possible language-specific pronunciation patterns.

### The need for authentic (induced) rather than enacted (posed) emotions

Acted vocal emotions differ from authentic ones. Not only are acted emotions perceived as stronger than their non-acted counterparts (Shahid et al., 2008), they also differ in their voice quality and reveal a more variable F0-contour (Jürgens et al., 2011). Thus, inducing rather than enacting emotions in stimuli serves to increase the ecological validity of studies in which these stimuli are used. We also considered that ecological validity should benefit from choosing non-actors as speakers, and note that findings suggest that vocal emotional expressions by professional actors also may not be better classified in emotion research than those by non-actors (Jürgens et al., 2015; Phatak et al., 2021). A meta-analysis which comparatively evaluated the effectiveness and validity of 11 emotion induction procedures demonstrated that movie scenes or stories are the most effective way to induce both positive and negative emotions (Westermann et al., 1996; for details on other common emotion induction techniques, please see Brandstätter et al., 2018). Correspondingly, for emotion induction for the stimuli of JAVMEPS, we presented all speakers with movie scenes which we had selected based on Schaefer et al. (2010).

JAVMEPS offers the scientific community a freely available corpus of stimuli. These stimuli could not only be used to answer various basic research questions but also to investigate emotion perception in individuals with difficulties in emotion recognition (e.g., individuals with autism, or cochlear implant (CI) users). Moreover, stimuli of JAVMEPS could also be utilized for perceptual trainings targeted at people with difficulties in emotion recognition (e.g., Schweinberger & von Eiff, 2022). Here we included a few CI users in the classification study of JAVMEPS, to allow first insights into how they perceive the present stimuli.^1^ In this paper, we describe the creation, validation, and usage of the JAVMEPS stimulus set.

## Materials and Methods

### Recording

#### Speakers

The JAVMEPS includes substantial recordings from 12 speakers (six female, six male). All speakers were native German speakers aged between 20 and 30 years (*M* = 24.25). Please see Table S2 (Supplemental Material) for detailed speaker information.

#### Procedure

In broad analogy to earlier auditory-only emotion research (e.g., Frühholz et al., 2015; von Eiff et al., 2022b) we created audiovisual recordings for four different bisyllabic, five-letter, and phonetically balanced pseudowords (/belam/, /namil/, /molen/, /loman/) in seven emotions (anger, fear, surprise, sadness, disgust, happiness, plus neutral) from each of the speakers. The recordings were obtained in a quiet and semi-anechoic room. Audio and video recordings were obtained simultaneously. Speakers sat on a chair in front of a green background and were illuminated by a four-point lighting system (Fig. S1, Supplemental Material). To standardize visual stimuli, they were asked to wear contact lenses instead of glasses, if required. Before recording, all speakers filled in a consent form and a self-report questionnaire on demographic data (including information on body height, weight, regional accents, experience with acting, and smoking habits). We then presented recording instructions on a notebook placed in front of the speakers. We asked speakers to pronounce all pseudowords clearly and distinctly, and in the same way a pre-recorded robotic model voice, which was given as an example did. We emphasized, however, that speakers should just take the robotic model voice as an orientation towards intonation and duration of each pseudoword, and that they should not try to imitate the robotic model voice, but speak with their natural voice. We moreover asked speakers to always directly face and directly look into the camera. Recording sessions consisted of seven blocks, one per emotion. Each block started with the option to listen to all four pseudowords, as a reminder how to pronounce each pseudoword. We then presented a movie scene to induce a certain emotion. Please refer to Table S1 (Supplemental Material) for a list of movie scenes we used for emotion induction. After watching the corresponding movie scene, speakers spoke the pseudowords. We recorded each pseudoword several times (about six times, or more in case the speaker had to cough, there was some background noise, etc.) to ensure that there were enough recordings to choose from. Speakers were encouraged to take self-paced breaks after each block and to drink still water whenever needed.

#### Technical information

We recorded voices with a Sennheiser MD 421-II microphone with a pop protection and a Zoom H4n audio interface (16-bit resolution, mono, 48 kHz). The audio interface was connected to a computer in the neighboring room at which the audio manager monitored the recordings via Adobe Audition 3.0.

### Post-processing and standardization of recordings

The best recordings of each pseudoword (in terms of artifacts, background noise, and clear pronunciation) were chosen. We extracted the audio recordings of the videos and saved them as .wav files with XMedia Recode freeware. We then time-synchronized the microphone audio files with the video audio files, using Praat (Boersma & Weenink, 2018). After this, using VirtualDub freeware, we combined the microphone audio and the video files and deinterlaced. Utilizing Adobe™ Premiere Pro CS6, we then replaced the green background of the videos with a black background, put a black mask over all videos, and cut the stimuli (12 frames before onset and 12 frames after offset). We then exported the files as auditory-only files (sample rate 48,000 Hz, Mono, 16 Bit), and normalized them to 70 dB with Praat. After that, we combined these normalized audio files with the silent videos, using Adobe™ Premiere Pro CS6. We then ensured that the sound intensity of these combined files was the same as before. If the peak amplitudes of the audio files differed, we redid combining the silent videos with the normalized audio files using different settings. The resulting files constitute Stimulus Set A of JAVMEPS.

To create stimuli of Stimulus Set B and Stimulus Set C of JAVMEPS, we applied a voice morphing approach to selected stimuli of (A), using the speech analysis, modification, and resynthesis framework TANDEM-STRAIGHT (Kawahara et al., 2008, 2013; Kawahara & Skuk, 2019). The selection of the stimuli of (A) which we used for creating Stimulus Set B and Stimulus Set C of JAVMEPS was based on the classification study described in the section “Validation”. STRAIGHT-based morphing generates highly natural-sounding synthesized voices (for further information, cf. Kawahara & Skuk, 2019; Skuk & Schweinberger, 2014). We systematically manipulated the acoustic parameters F0, aperiodicity (AP), formant frequencies (FF), spectrum level (SL), and time (T). AP, FF, and SL were considered to reflect timbre, in line with previous research (Skuk & Schweinberger, 2014). Utilizing Praat, we normalized all resulting audio files to 70 dB. To create the AV stimuli of (C) of JAVMEPS, we combined the normalized audio files with the silent videos, using Premiere Pro CS6 (for details on the synchronization procedure via temporal morphing, please see Supplemental Material, section 4, and Table S3). In line with the procedure for AV stimuli of (A), we made sure that the sound intensity of these combined files was the same as before and repeated combining audio and video files if the sound intensity differed.

Furthermore, we masked 480 ms before voice onset and after voice offset in all visual-only and AV stimuli by a sophisticated masking algorithm to ensure equal length of audible voice and visible dynamic face information (for more information on the masking algorithm, including the full script, please refer to [https://osf.io/r3xqw/]).

### Description of JAVMEPS files

JAVMEPS comprises 2256 stimulus files (disregarding two different - grey or black - versions of video masking), which can be freely downloaded on OSF [https://osf.io/r3xqw/]. The stimulus folders on OSF also contain a detailed description of filenames of all stimuli of JAVMEPS. Moreover, note that each JAVMEPS folder which contains auditory-only stimuli also contains detailed information on acoustic characteristics of each auditory-only stimulus.

**Fig. 1:**
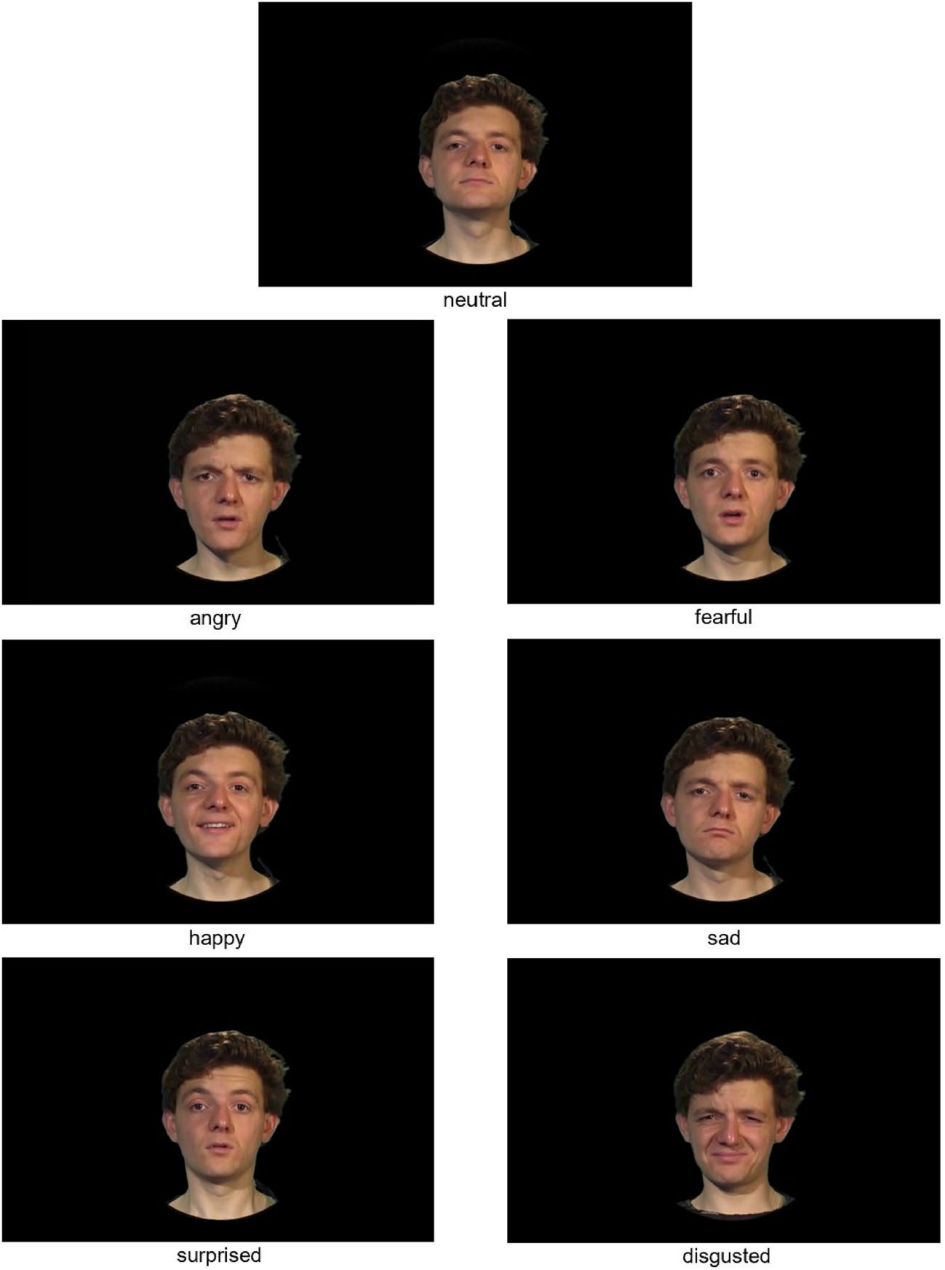
Still-image frame examples of the seven emotions contained in JAVMEPS

Stimulus Set A of JAVMEPS (1008 stimuli) contains recordings of 12 speakers (six female, six male) who speak four pseudowords (/belam/, /namil/, /molen/, /loman/) with six naturalistic basic emotions (anger, fear, happiness, disgust, sadness, surprise) plus a neutral emotion, in an auditory-only, a visual-only, and a congruent AV condition. Figure 1 illustrates still-image frame examples of each of the emotional expressions contained in JAVMEPS. Recordings of eight speakers (four female, four male), uttering two pseudowords (/namil/, /loman/), constitute Stimulus Set B of JAVMEPS (288 stimuli). This part consists of vocal caricatures (140%), original voices (100%), and vocal anti-caricatures (60%) between neutral and happy, fearful, angry, sad, disgusted, or surprised voices. Stimulus Set C of JAVMEPS (960 stimuli) comprises precisely time-synchronized congruent and incongruent AV (and the corresponding auditory-only) stimuli, spoken by eight speakers (four female, four male) with two emotions (anger, surprise). This part of JAVMEPS offers stimuli with original vocal emotional intensity (C1) and with graded congruence of AV expressions (C2), from vocal emotion caricatures over original voices to vocal emotion anti-caricatures (140, 120, 100, 80, or 60%).

Note that for realizing this graded congruence of AV expressions in part (C2) of JAVMEPS, we systematically manipulated individual acoustic parameters along surprised–angry and angry–surprised morph continua. Whereas (C1) comprises four pseudowords (/belam/, /namil/, /molen/, /loman/), (C2) contains two pseudowords (/belam/, /molen/).

### Validation

#### Statistical analysis

Statistical analyses were performed using R (R Core Team, 2020). Trials with reaction times > 12,000 ms (from stimulus offset; 0.47% of experimental trials) were excluded. We performed Epsilon corrections for heterogeneity of covariances (Huynh & Feldt, 1976) where appropriate.

#### Participants in classification study

Twenty-two (17 female, five male) normal-hearing listeners, aged between 20 and 31 years (*M* = 24.82), and four (two female, two male) CI users, aged between 22 and 72 years (*M* = 52.25), contributed data to the classification study; please note that one further normal-hearing listener participated in the classification study but we excluded the data right after her testing session, as she recognized a speaker as personally familiar. We included a few CI users because they represent people for whom the perception of vocal emotions is particularly challenging (e.g., Agrawal et al., 2013; Jiam et al., 2017; Kim & Yoon, 2018; Luo et al., 2007; Paquette et al., 2018; Schorr et al., 2009; von Eiff et al., 2022a, b). Normal-hearing listeners were tested at the Friedrich Schiller University Jena; CI users were tested at the Cochlear Implant Rehabilitation Centre in Erfurt. All participants were native German speakers without neurological or psychiatric diagnoses. CI users reported no other otologic disorders and had either bilateral implants or unilateral implants and a diagnosis of severe to profound hearing loss in the non-implanted ear. Normal-hearing listeners did not report any hearing difficulties. None of the participants who contributed data had taken part in stimulus creation or reported prior familiarity with any of the speakers used in the experiment.

#### Stimuli

We selected recordings of 12 speakers (six female, six male), speaking two pseudowords (/belam/, /molen/) with six naturalistic basic emotions (anger, fear, happiness, disgust, sadness, surprise) plus a neutral emotion, in an auditory-only, a visual-only, and a congruent AV condition. These stimuli (i.e., half of all stimuli of Stimulus Set A of JAVMEPS) correspond to the original stimuli we used for creating the other stimuli of JAVMEPS (i.e., stimuli of (B) and (C) of JAVMEPS), including vocal caricatures, anti-caricatures, and the precisely time-synchronized congruent and incongruent AV stimuli with varying congruence of AV expressions. Altogether, we presented 504 stimuli (12 speakers × 7 emotions × 2 pseudowords × 3 conditions) in the classification study. Mean stimulus duration was 1693.8 ms (*SD* = 123.7 ms, range: 1440–2160 ms).

#### Experimental setting

All participants performed the experiment using the same technical equipment, including a Fujitsu™ LIFEBOOK E754 notebook with a 32-bit operating system, an Intel Core i5-4210M CPU processor (2.60 GHz), 1.600 MHz, 500 GB/8 GB SSD-Cache, and a 39.6 cm (15.6’’) HD display. We presented voice stimuli binaurally in mono at a peak intensity of approximately 70 dB(A) SPL, as measured with a Brüel and Kjær™ Precision Sound Level Meter Type 2206, using two Creative™ Inspire T10 loudspeakers (5 Watts RMS per channel, two channels, 80 Hz ~ 20 kHz). All participants were tested individually in a sound-attenuated chamber (~ 4 m^2^), with 1 m between head and monitor, with loudspeakers placed next to both monitor sides.

#### Procedure

Experimental sessions lasted about 40 min. All participants filled in a self-report questionnaire on demographic data. We then presented a computer experiment programmed with E-Prime® 3.0. For its duration, CI users used the same CI processor(s) as in their daily routines. Unilateral CI users were asked to turn off any hearing aids in the contralateral ear to avoid the contribution of residual hearing. Participants performed a seven-alternative forced choice (7-AFC) task to indicate anger, fear, happiness, disgust, sadness, surprise, or a neutral emotion. Experimental instructions were presented in writing on the computer screen to avoid interference from the experimenter’s voice. We asked participants to focus carefully on each stimulus and to decide as accurately and as fast as possible whether they perceived it as rather angry, fearful, happy, disgusted, sad, surprised, or neutral. Responses were made by clicking a button with the computer mouse. No feedback on accuracy was given. Experimental trials were presented in four blocks of 126 trials each. Self-paced breaks were allowed after each block. All stimuli were presented once in random order. Each trial started with a green fixation cross which, after 500 ms, was replaced by either a silent video, an AV congruent stimulus, or a green question mark, together with a green loudspeaker symbol. The onset of the question mark, together with the loudspeaker symbol, coincided with the onset of an auditory-only stimulus and remained on screen until the offset of the stimulus.

## Results

Emotion category ratings were coded as correct when the category which a participant selected in the classification study matched the category which the presented movie scene in the recording session was supposed to elicit in the speaker. If the category selected in the classification study did not match the intended category, it was coded as incorrect.

### Accuracy measures

Normal-hearing individuals showed good classification performance for auditory-only (*M*_corrA_ = .517, *SD* = .500) and very good classification performance for visual-only (*M*_corrV_ = .676, *SD* = .468) and AV (*M*_corrAV_ = .764, *SD* = .425) stimuli.^2^ For emotions, across modalities, classification rates of NH individuals for fearful (*M*_corrfearful_ = .473, *SD* = .499) stimuli were good. NH raters showed very good classification performance for disgusted (*M*_corrdisgusted_ = .601, *SD* = .500), sad (*M*_corrsad_ = .648, *SD* = .478), angry (*M*_corrangry_ = .651, *SD* = .477), and neutral (*M*_corrneutral_ = .687, *SD* = .464) stimuli, and excellent classification rates for surprised (*M*_corrsurprised_ = .752, *SD* = .432) and happy (*M*_corrhappy_ = .756, *SD* = .430) stimuli. In contrast, unsurprisingly, CI users showed lower classification rates for auditory-only stimuli (*M*_corrA_ = .232, *SD* = .423). Importantly, they, however, performed clearly above chance levels of .14. The classification performance of CI users for visual-only (*M*_corrV_ = .511, *SD* = .500)^3^ and AV (*M*_corrAV_ = .511, *SD* = .500) stimuli was good. Across modalities, CI users’ classification rates were appropriate for disgusted (*M*_corrdisgusted_ = .234, *SD* = .424), fearful (*M*_corrfearful_ = .312, *SD* = .464), and sad (*M*_corrsad_ = .395, *SD* = .490) stimuli; they were good for angry (*M*_corrangry_ = .431, *SD* = .496), surprised (*M*_corrsurprised_ = .451, *SD* = .498), neutral (*M*_corrneutral_ = .538, *SD* = .499), and happy (*M*_corrhappy_ = .564, *SD* = .497) stimuli.

**Fig. 2:**
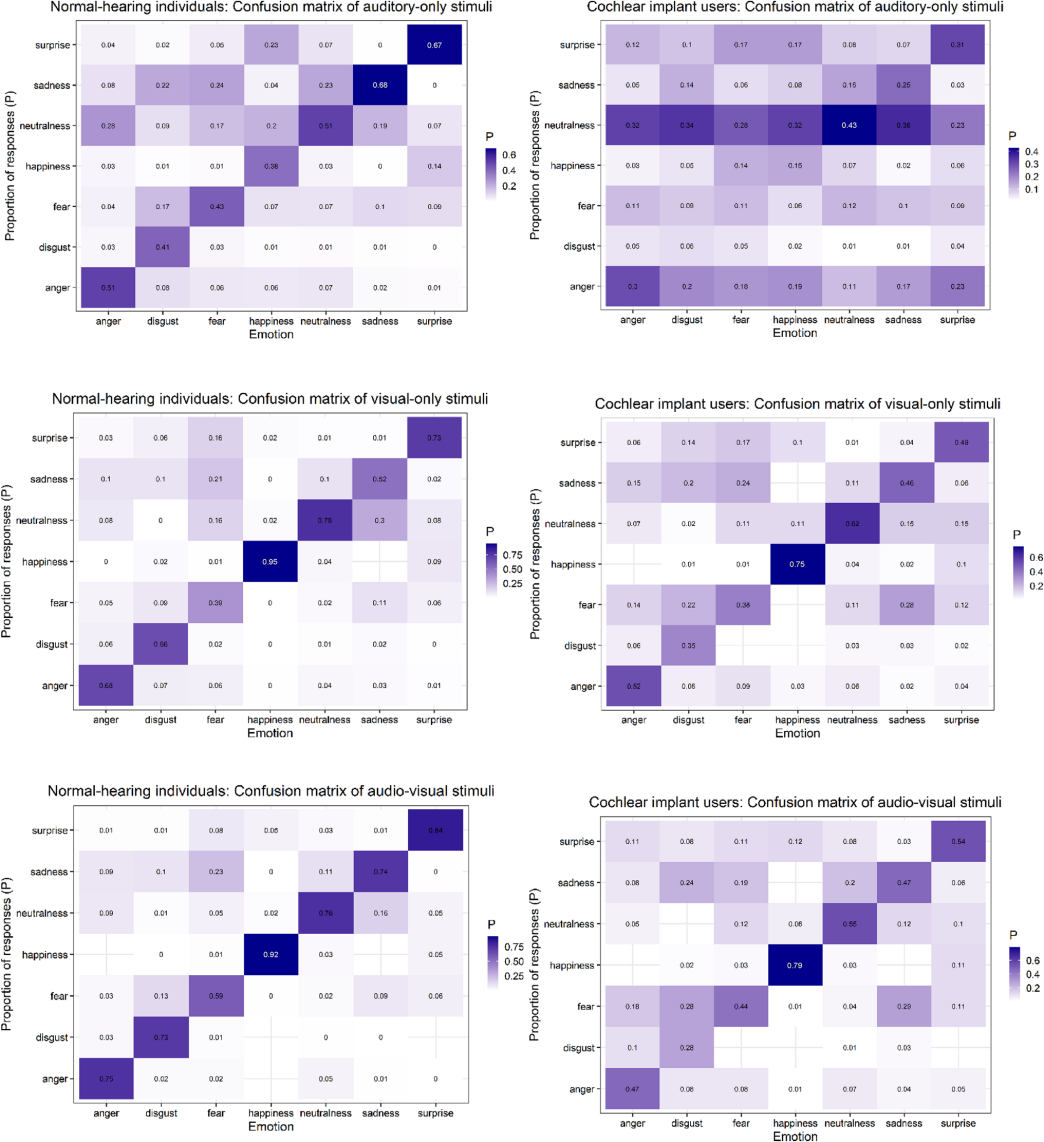
Classification performance of normal-hearing individuals and CI users for the seven emotions contained in JAVMEPS, separately for auditory-only, visual-only, and AV stimuli. Note different scaling across plots in the interest of visibility

Figure 2 shows details on classification performance of both NH raters and CI users for each emotion, separately for each modality. NH individuals showed classification rates for each emotion in the auditory-only condition of at least ≥ .38 correct, in the visual-only condition ≥ .39 correct, and in the AV condition ≥ .59 correct. CI users showed classification rates for each emotion in the auditory-only condition ≥ .06 correct [with best classification rates for neutralness (.43), surprise (.31), and anger (.30)], in the visual-only condition ≥ .35 correct, and in the AV condition ≥ .28 correct.

### Interrater reliability

We estimated the degree of agreement between the emotion category responses of normal-hearing participants in the classification study. Since we analyzed ratings for more than two categorical variables, we assessed interrater reliability with Fleiss’ kappa, which is recommended for inter-rater agreement between more than two raters. The Kappa scores reflect the degree of agreement in classification over that which would be expected by chance. Kappa values < 0 show poor agreement, .01 to .20 slight agreement, .21 to .40 fair agreement, .41 to .60 moderate agreement, .61 to .80 substantial agreement, and .81 to 1 indicate (nearly) perfect agreement (Landis & Koch, 1977). We assessed interrater reliability across both modality and emotion. The Fleiss kappa scores for auditory-only (kappa = .304), visual-only (kappa = .316), and AV (kappa = .274) stimuli showed fair agreement between all participants in the classification study across modality.

Across emotion, the degree of agreement differed; whereas there was slight agreement for neutral (kappa = .142) and surprised (kappa = .158) stimuli, and fair agreement for disgusted (kappa = .342), fearful (kappa = .287), and sad (kappa = .222) stimuli, the agreement for angry (kappa = .485) and happy (kappa = .498) stimuli was moderate. All calculated kappa values were significantly different from zero (all *p*s ≤ .0001), indicating that the observed interrater agreement rates were not due to chance.

## Discussion

In this paper, we present a novel face and voice database – the JAVMEPS stimulus set, which is designed for experimental research on both unimodal face and unimodal voice processing of emotions, as well as for the processing of AV face-voice cues. JAVMEPS contains various standardized high-quality dynamic face and voice samples which are freely available for the scientific community via the following link: https://osf.io/r3xqw/.

It meets diverse requirements which are crucial for emotion recognition research, such as a systematically varied emotion intensity in the stimuli with state-of-the-art voice morphing technology, and the use of emotion induction in the speaker’s recording sessions.

When producing stimulus sets, inducing rather than enacting emotions increases the ecological validity of stimuli, since acted vocal emotions differ from authentic vocal emotions (Jürgens et al., 2011; Shahid et al., 2008). Notably, however, inducing emotions by presenting films or music, or using imagery methods or stressor scenarios, could produce relatively weak effects, and there may be uncertainty as to the emotion that was elicited (Laukka et al., 2011; Tcherkassof et al., 2007). Movie scenes – i.e., the utilized emotion induction technique for JAVMEPS – or stories were shown to be the most effective way to induce emotions (Westermann et al., 1996). The classification study of JAVMEPS provided evidence that, in fact, emotion induction worked successfully for this stimulus set.

One possible point for criticism on stimulus sets in which emotion induction was used addresses the issue of rather small sample sizes. Instead of conducting elaborate speaker recording sessions, which may result in fewer speakers, one could compile a stimulus set of seemingly spontaneous emotion expressions “in the wild” (e.g., Dawel et al., 2022), for instance by cutting scenes from TV shows, e.g., quiz shows or late-night talk shows. Producing such stimulus sets would possibly also result in more ecological valid stimuli than stimuli showing only enacted emotions. However, one could usually only design a stimulus set in a between-subject design of speakers, instead of a within-subject design – which results in various disadvantages in designs of experiments in which such stimuli are used. In naturalistic stimulus sets, individuals often appear in very few clips, and the recording situation, material, and quality can vary profoundly (Scherer, 2003). Thus, these stimuli can generally not be as standardized, and, therefore, provide a comparable degree of experimental control, compared to the present stimuli for which environmental aspects (lighting, recording equipment, background, spoken (pseudo)words, etc.) were kept constant. Crucially, the present recordings allowed us to produce precisely time-synchronized AV stimuli with incongruent facial and vocal emotions which differ only in emotion congruence from the corresponding congruent AV stimuli. Furthermore, induced emotional expressions are intended to communicate a single emotional category (e.g., “anger”), whereas raters frequently give mixed labels to naturalistic recordings (Cowie, 2009). Accordingly, we believe that the advantages of sets which use emotion induction, compared to sets which use “in the wild” expressions, outweigh the disadvantages (cf. Livingstone & Russo, 2018, for a similar argument).

It is important to note that pronunciation of pseudowords in JAVMEPS followed German pronunciation rules. According to the so-called dialect theory, emotional communication is culturally universal but characterized by accents which reflect the distinct cultural style for expressing nonverbal cues (Elfenbein, 2013). The theory argues that people assess emotional expressions based on their own learned expressive style, even when emotional expressions are posed by another culture. Correspondingly, the impact of diverse accents on emotion recognition will vary in accordance with the mismatch between the emotional cues expressed in one culture and the own cultural style. Jiang and colleagues (Jiang et al., 2015) studied the nature of in-group advantages in vocal emotion recognition by presenting pseudoutterances, expressed in English and Hindi, to English and Hindi listeners. They found that in each language condition, native listeners were more accurate and faster to recognize emotions, compared to non-native listeners. Thus, in line with dialect theory, the authors showed that nonverbal dialects impede the accuracy and the efficiency of vocal emotion processing in cross-cultural settings. Pell et al. (2009) compared how monolingual speakers of Argentine Spanish recognize emotions from pseudo-utterances produced in their native language and in English, German, and Arabic. The authors showed that vocal emotion recognition exceeded chance levels in each language condition, even though Spanish listeners performed better in their native language. Applied to the present case, we cannot exclude the possibility that non-German perceivers recognize stimuli of JAVMEPS slower and less accurately than German perceivers. Although we believe that any such cross-cultural effects should be minimal as long as JAVMEPS is used in a culture which is similar to German in terms of emotion expression, this issue deserves further exploration. Irrespective of this, we argue that JAVMEPS can be used across a large range of languages and cultures in studies in which the focus is on group differences of participants in emotion recognition, and as long as the participants being compared are from the same culture.

Validation of JAVMEPS was performed with 22 normal-hearing, healthy individuals, and, innovatively, four CI users, who represent people facing challenges in vocal emotion recognition (e.g., Agrawal et al., 2013; Jiam et al., 2017; Kim & Yoon, 2018; Luo et al., 2007; Paquette et al., 2018; Schorr et al., 2009; von Eiff et al., 2022a, b). Note that the good classification performance that NH individuals showed in the classification study, with rating scores around 52% for auditory-only, 68% for visual-only, and 76% for AV stimuli, is comparable to classification study results of other published stimulus sets comprising full sentences as AV emotional stimuli. Examples include the CREMA-D (Cao et al., 2014) and the RAVDESS (Livingstone & Russo, 2018). Whereas the CREMA-D achieved 41% for auditory-only, 58% for visual-only, and 64% for AV stimuli, the RAVDESS achieved rating scores of 60% for auditory-only, 75% for visual-only, and 80% for AV stimuli. Classification performance of CI users in our classification study, however, was lower. This is unsurprising, given that some CI users actually perform at chance levels when recognizing emotions, even in 2-AFC emotion recognition tasks (von Eiff et al., 2022a, b). Importantly, CI users recognized the auditory-only stimuli of JAVMEPS clearly above chance levels, though – with recognition rates around 23%. CI users’ recognition rates of auditory-only stimuli for the emotions surprise and anger were even at 30% and 31%, respectively. In fact, surprise and anger are the two emotions contained in part (C) of JAVMEPS. Thus, the JAVMEPS stimulus set is not only highly qualified for emotion recognition research in NH individuals, but also offers validated choices of stimulus material in emotion recognition research in CI users. Researchers who consider the use of JAVMEPS in populations with difficulties in vocal emotion perception are referred to the possibility to use caricatured emotions in the database, which were recently demonstrated to enhance vocal emotion recognition with a CI (von Eiff et al., 2022a).

### Supplementary Information

Below is the link to the electronic supplementary material.

Supplementary file1 (DOCX 111 KB)

## Data Availability

All data are available at OSF [https://osf.io/r3xqw/]. Note that the repository contains data, materials and scripts that will enable users to select classification-based stimulus subsets according to individual study plans where desired.

## Footnotes

1 Note that a subset of the stimuli of the JAVMEPS was also successfully used to investigate audiovisual integration in vocal emotion perception by a larger sample of CI users (von Eiff et al., 2022a).

2 To put these results into perspective, please consider validation results of other published stimulus sets comprising full sentences as AV emotional stimuli, e.g., the CREMA-D (Cao et al., 2014) with 41% for auditory-only, 58% for visual-only, and 64% for AV stimuli, and the RAVDESS (Livingstone & Russo, 2018) with 60% for auditory-only, 75% for visual-only, and 80% for AV stimuli.

3 We caution against overinterpreting absolute performance differences for visual-only stimuli between CI users and hearing listeners in view of the very small (*N* = 4) sample of CI users. Nevertheless, it may be worth mentioning that the present data correspond to a study on emotion recognition in CI users, which suggested a reduction of emotional sensitivity to visual facial expressions in adult CI users (Ambert-Dahan et al., 2017).

## Code availability

Relevant R scripts and code is available at OSF [https://osf.io/r3xqw/].
