# Supplementary material for: The Jena Audiovisual Stimuli of Morphed Emotional Pseudospeech (JAVMEPS): A database for emotional auditory-only, visual-only, and congruent and incongruent audiovisual voice and dynamic face stimuli with varying voice intensities
Source: Behav Res Methods. 2023 Oct 11;56(5):5103–15. doi: 10.3758/s13428-023-02249-4 (PMC11289065; doi:10.3758/s13428-023-02249-4)
Supplement: Supplementary file 1 — Supplementary file1 (DOCX 111 KB) [file 13428_2023_2249_MOESM1_ESM.docx]

**The Jena Audiovisual Stimuli of Morphed Emotional Pseudospeech (JAVMEPS):
A database for emotional auditory-only, visual-only, and
congruent and incongruent audiovisual voice and dynamic face stimuli
with varying voice intensities**

Celina I. von Eiff^1,2,3,4^, Julian Kauk^1^, and Stefan R. Schweinberger^1,2,3,4^

**Supplemental Material**

# **1 Physical setup of the recording studio**

Speakers were recorded individually in a professional recording studio. The physical layout of the recording studio is shown in the following figure.


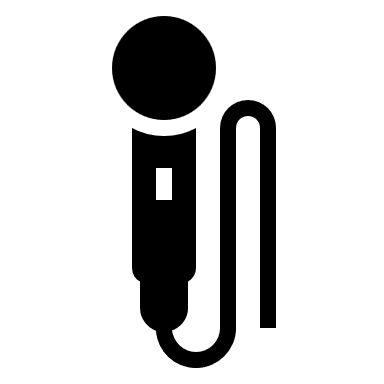

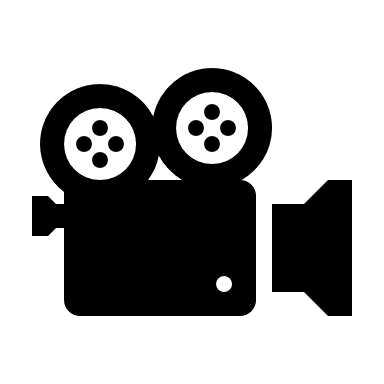


*Light*

*Video camera*

*Notebook*

*Light*

*Microphone*

*Speaker*

*Light*

*Green Screen*

*Light*

*Figure S1.* Physical layout of the recording studio used to record stimuli of JAVMEPS.

# **2 Movie scenes used for emotion induction**

For emotion induction for stimuli from JAVMEPS, we presented all speakers with movie scenes which we selected based on Schaefer, Nils, Sanchez, & Philippot (2010).

Table S1. List of Movies selected for Emotion Induction.

| **Movie** | **Scene** | **Emotion we aimed to induce** |
| --- | --- | --- |
| Schindler’s List | A concentration camp commander randomly shoots prisoners from his balcony. | Anger |
| The Blair Witch Project | Final scene in which the characters are apparently killed. | Fear |
| City of Angels | Maggie (Meg Ryan) dies in Seth’s (Nicolas Cage) arms. | Sadness |
| Trainspotting | The main character dives into a filthy toilet. | Disgust |
| Skyfall* | James Bond (Daniel Craig) talks to his opponent, when, suddenly, a subway comes through the ceiling. | Surprise |
| Intouchables* | At the end of his birthday party, Philippe (François Cluzet) educates Driss (Omar Sy) on famous classical pieces; Driss then plays music he enjoys, livening up the party. | Happiness |
| Animal documentary* | Perceiver sees different animals whereas a speaker talks about animals’ body clocks. | Neutral expression |

*Please note that Schaefer et al. did not specifically suggest these movies but that we selected them inspired by their selection.

# **3 Characteristics of speakers**

Please note that one speaker (MK06) agreed that her recordings will be part of a stimuli database and will be used by other researchers but did not agree to provide her recordings for publication in scientific media and for teaching purposes. All other speakers agreed to this.

| **MB10** |  | February 27th 2019 | m | 30 | 1988 | r | 180 | 66 | Scientific researcher | / | / | / |  | German | English (2)  French (5)  Spanish (5) | None |  | 1 | 1 | 1 | 6 | 5 | Smoker between 2005-2008 | Table S2. Detailed Speaker Information. Note: m, male; f, female; r, right; Height in centimeters; weight in kilogram; foreign language is specified within a niveau between 1, fluent, and 5, not at all; factors which influence the voice are assessed on a scale 1, completely right, to 6, completely false |
| --- | --- | --- | --- | --- | --- | --- | --- | --- | --- | --- | --- | --- | --- | --- | --- | --- | --- | --- | --- | --- | --- | --- | --- | --- |
| **LF03** |  | February 7th 2019 | f | 21 | 1997 | r | 166 | 65 | University student | Psychology | FSU Jena | 2016 |  | German | English (1)  Portuguese (2)  Spanish (3)  French (4) | Hessian |  | 2 | 2 | 1 | 6 | 4 | Current smoker (2-3 cigarettes per day) |  |
| **FR02** |  | February 6th 2019 | f | 23 | 1995 | r | 176 | 64 | University student | Psychology | FSU Jena | 2015 |  | German | English (1) | None |  | 2 | 2 | 1 | 6 | 3 | Lifetime non-smoker |  |
| **JJ04** |  | February 12th 2019 | m | 25 | 1993 | r | 188 | 88 | University student | Education (English, Business) | FSU Jena | 2015 |  | German | English (1)  French (6) | None |  | 2 | 2 | 3 | 6 | 5 | Lifetime non-smoker |  |
| **HO05** |  | February 12th 2019 | f | 22 | 1996 | r | 163 | 58 | University student | Medicine | FSU Jena | 2016 |  | German | Spanish (1)  English (1)  French (3) | None |  | 4 | 2 | 1 | 6 | 6 | Lifetime non-smoker |  |
| **MK06** |  | February 12th 2019 | f | 20 | 1998 | r | 173 | 67 | University student | Computer science | FSU Jena | 2017 |  | German | English (2)  French (5) | None |  | 3 | 4 | 3 | 6 | 3 | Lifetime non-smoker |  |
| **JW07** |  | February 13th 2019 | f | 27 | 1991 | r | 171 | 65 | University student | Medicine | FSU Jena | 2017 |  | German | English (2) | None |  | 6 | 5 | 4 | 6 | 3 | Lifetime non-smoker |  |
| **LN08** |  | February 14th 2019 | f | 23 | 1995 | r | 170 | 60 | University student | Speech Science, German | FSU Jena | 2015 |  | German | English (1)  Spanish (2)  Italian (2) | None |  | 1 | 1 | 1 | 1 | 5 | Smoker between 2012-2016 |  |
| **BZ09** |  | February 20th 2019 | m | 25 | 1993 | r | 182 | 87 | University student | Education | FSU Jena | 2016 |  | German | English (1)  Spanish (2) | None |  | 6 | 4 | 5 | 6 | 5 | Lifetime non-smoker |  |
| **LG11** |  | February 28th 2019 | m | 24 | 1994 | r | 174 | 70 | University student | Education (Geopraphy, Sport) | FSU Jena | 2013 |  | German | English (3)  French (6) | None |  | 3 | 3 | 3 | 4 | 4 | Lifetime non-smoker |  |
| **TL12** |  | May 23rd 2019 | m | 26 | 1992 | r | 170 | 70 | University student | Medicine | FSU Jena | 2011 |  | German | English (1)  Polish (2)  Spanish (4)  French (5) | None |  | 4 | 5 | 4 | 5 | 3 | Lifetime non-smoker |  |
| **WK01** |  | December 20th 2018 | m | 25 | 1993 | r | 185 | 90 | University student | Business | FSU Jena | 2017 |  | German | English (2)  French (4) | None |  | 3 | 2 | 4 | 1 | 3 | Lifetime non-smoker |  |
| **Speaker** | **General Information** | Date of Recording | Gender | Age at Recording | Year of Birth | Handedness | Height | Weight | **Occupation** | Subject | University | since | **Language skills** | Native language | Foreign language(s) (niveau) | Dialect | **Voice influencing factors** | Practiced speaker | Free speech experience | Experience in reading | Theater acting experiences | Excitement to speak | Smoking behavior |  |

# **4 Synchronization of stimuli of Part (C): Time anchor points**

Part (C) of JAVMEPS comprises precisely time-synchronized congruent and incongruent AV stimuli, with original vocal emotional intensity (C1) and with graded congruence of AV expressions (C2). We used 12 time anchor points of congruent vocal emotions to inform temporal morphing of incongruent vocal emotions towards the identical time structure.

Table S3. Time Anchor Points of Auditory Stimuli in Part (C) of JAVMEPS (in seconds).

| Pseudoword | Speaker | Vocal and Facial Emotion | TA 1 | TA 2 | TA 3 | TA 4 | TA 5 | TA 6 | TA 7 | TA 8 | TA 9 | TA 10 | TA 11 | TA 12 |
| --- | --- | --- | --- | --- | --- | --- | --- | --- | --- | --- | --- | --- | --- | --- |
| belam | WK01 | VA FA (cong) | 0.51 | 0.526 | 0.592 | 0.664 | 0.71 | 0.753 | 0.793 | 0.83 | 0.866 | 0.903 | 1.018 | 1.149 |
|  |  | VS FA (incong) | 0.51 | 0.526 | 0.592 | 0.664 | 0.71 | 0.753 | 0.793 | 0.83 | 0.866 | 0.903 | 1.018 | 1.149 |
|  |  | VS FS (cong) | 0.545 | 0.554 | 0.61 | 0.664 | 0.698 | 0.732 | 0.77 | 0.805 | 0.841 | 0.88 | 0.924 | 0.97 |
|  |  | VA FS (incong) | 0.545 | 0.554 | 0.61 | 0.664 | 0.698 | 0.732 | 0.77 | 0.805 | 0.841 | 0.88 | 0.924 | 0.97 |
| belam | HO05 | VA FA (cong) | 0.543 | 0.562 | 0.613 | 0.663 | 0.703 | 0.743 | 0.808 | 0.876 | 0.943 | 1.009 | 1.089 | 1.167 |
|  |  | VS FA (incong) | 0.543 | 0.562 | 0.613 | 0.663 | 0.703 | 0.743 | 0.808 | 0.876 | 0.943 | 1.009 | 1.089 | 1.167 |
|  |  | VS FS (cong) | 0.521 | 0.587 | 0.636 | 0.686 | 0.732 | 0.775 | 0.839 | 0.899 | 0.962 | 1.024 | 1.11 | 1.196 |
|  |  | VA FS (incong) | 0.521 | 0.587 | 0.636 | 0.686 | 0.732 | 0.775 | 0.839 | 0.899 | 0.962 | 1.024 | 1.11 | 1.196 |
| belam | MK06 | VA FA (cong) | 0.533 | 0.548 | 0.607 | 0.67 | 0.715 | 0.755 | 0.811 | 0.871 | 0.928 | 0.988 | 1.053 | 1.121 |
|  |  | VS FA (incong) | 0.533 | 0.551 | 0.635 | 0.72 | 0.768 | 0.811 | 0.872 | 0.933 | 0.992 | 1.052 | 1.145 | 1.234 |
|  |  | VS FS (cong) | 0.533 | 0.551 | 0.635 | 0.72 | 0.768 | 0.811 | 0.872 | 0.933 | 0.992 | 1.052 | 1.145 | 1.234 |
|  |  | VA FS (incong) | 0.533 | 0.548 | 0.607 | 0.67 | 0.715 | 0.755 | 0.811 | 0.871 | 0.928 | 0.988 | 1.053 | 1.121 |
| belam | JW07 | VA FA (cong) | 0.516 | 0.54 | 0.649 | 0.763 | 0.796 | 0.831 | 0.906 | 0.983 | 1.057 | 1.129 | 1.196 | 1.26 |
|  |  | VS FA (incong) | 0.516 | 0.54 | 0.649 | 0.763 | 0.796 | 0.831 | 0.906 | 0.983 | 1.057 | 1.129 | 1.196 | 1.26 |
|  |  | VS FS (cong) | 0.528 | 0.552 | 0.6 | 0.653 | 0.684 | 0.713 | 0.746 | 0.782 | 0.819 | 0.854 | 0.942 | 1.028 |
|  |  | VA FS (incong) | 0.528 | 0.552 | 0.6 | 0.653 | 0.684 | 0.713 | 0.746 | 0.782 | 0.819 | 0.854 | 0.942 | 1.028 |
| belam | LN08 | VA FA (cong) | 0.509 | 0.532 | 0.58 | 0.625 | 0.681 | 0.736 | 0.805 | 0.874 | 0.939 | 1.003 | 1.13 | 1.253 |
|  |  | VS FA (incong) | 0.509 | 0.532 | 0.58 | 0.625 | 0.681 | 0.736 | 0.805 | 0.874 | 0.939 | 1.003 | 1.13 | 1.253 |
|  |  | VS FS (cong) | 0.539 | 0.555 | 0.594 | 0.631 | 0.664 | 0.69 | 0.768 | 0.851 | 0.939 | 1.022 | 1.127 | 1.232 |
|  |  | VA FS (incong) | 0.539 | 0.555 | 0.594 | 0.631 | 0.664 | 0.69 | 0.768 | 0.851 | 0.939 | 1.022 | 1.127 | 1.232 |
| belam | MB10 | VA FA (cong) | 0.541 | 0.557 | 0.597 | 0.636 | 0.675 | 0.715 | 0.762 | 0.808 | 0.859 | 0.906 | 0.991 | 1.074 |
|  |  | VS FA (incong) | 0.541 | 0.557 | 0.597 | 0.636 | 0.675 | 0.715 | 0.762 | 0.808 | 0.859 | 0.906 | 0.991 | 1.074 |
|  |  | VS FS (cong) | 0.542 | 0.559 | 0.591 | 0.624 | 0.654 | 0.688 | 0.742 | 0.794 | 0.848 | 0.913 | 0.966 | 1.026 |
|  |  | VA FS (incong) | 0.542 | 0.559 | 0.591 | 0.624 | 0.654 | 0.688 | 0.742 | 0.794 | 0.848 | 0.913 | 0.966 | 1.026 |
| belam | LG11 | VA FA (cong) | 0.527 | 0.544 | 0.584 | 0.62 | 0.66 | 0.701 | 0.763 | 0.823 | 0.884 | 0.943 | 1.019 | 1.102 |
|  |  | VS FA (incong) | 0.527 | 0.544 | 0.584 | 0.62 | 0.66 | 0.701 | 0.763 | 0.823 | 0.884 | 0.943 | 1.019 | 1.102 |
|  |  | VS FS (cong) | 0.546 | 0.569 | 0.603 | 0.635 | 0.69 | 0.748 | 0.819 | 0.89 | 0.962 | 1.036 | 1.11 | 1.182 |
|  |  | VA FS (incong) | 0.546 | 0.569 | 0.603 | 0.635 | 0.69 | 0.748 | 0.819 | 0.89 | 0.962 | 1.036 | 1.11 | 1.182 |
| belam | TL12 | VA FA (cong) | 0.515 | 0.529 | 0.561 | 0.593 | 0.619 | 0.645 | 0.682 | 0.72 | 0.759 | 0.798 | 0.87 | 0.943 |
|  |  | VS FA (incong) | 0.515 | 0.529 | 0.561 | 0.593 | 0.619 | 0.645 | 0.682 | 0.72 | 0.759 | 0.798 | 0.87 | 0.943 |
|  |  | VS FS (cong) | 0.547 | 0.567 | 0.595 | 0.621 | 0.651 | 0.681 | 0.726 | 0.774 | 0.822 | 0.873 | 0.95 | 1.028 |
|  |  | VA FS (incong) | 0.547 | 0.567 | 0.595 | 0.621 | 0.651 | 0.681 | 0.726 | 0.774 | 0.822 | 0.873 | 0.95 | 1.028 |
| *belam* |  | *Mean* | *0.531* | *0.552* | *0.603* | *0.654* | *0.694* | *0.732* | *0.788* | *0.845* | *0.901* | *0.958* | *1.04* | *1.123* |
| molen | WK01 | VA FA (cong) | 0.526 | 0.62 | 0.7 | 0.771 | 0.861 | 0.95 | 1.012 | 1.075 | 1.143 | 1.21 | 1.364 | 1.523 |
|  |  | VS FA (incong) | 0.526 | 0.62 | 0.7 | 0.771 | 0.861 | 0.95 | 1.012 | 1.075 | 1.143 | 1.21 | 1.364 | 1.523 |
|  |  | VS FS (cong) | 0.53 | 0.635 | 0.702 | 0.769 | 0.829 | 0.901 | 0.957 | 1.012 | 1.068 | 1.126 | 1.214 | 1.301 |
|  |  | VA FS (incong) | 0.53 | 0.635 | 0.702 | 0.769 | 0.829 | 0.901 | 0.957 | 1.012 | 1.068 | 1.126 | 1.214 | 1.301 |
| molen | HO05 | VA FA (cong) | 0.539 | 0.618 | 0.69 | 0.761 | 0.797 | 0.833 | 0.884 | 0.935 | 0.992 | 1.048 | 1.112 | 1.177 |
|  |  | VS FA (incong) | 0.539 | 0.618 | 0.69 | 0.761 | 0.797 | 0.833 | 0.884 | 0.935 | 0.992 | 1.048 | 1.112 | 1.177 |
|  |  | VS FS (cong) | 0.539 | 0.576 | 0.626 | 0.677 | 0.706 | 0.737 | 0.792 | 0.854 | 0.914 | 0.976 | 1.073 | 1.163 |
|  |  | VA FS (incong) | 0.539 | 0.576 | 0.626 | 0.677 | 0.706 | 0.737 | 0.792 | 0.854 | 0.914 | 0.976 | 1.073 | 1.163 |
| molen | MK06 | VA FA (cong) | 0.539 | 0.587 | 0.669 | 0.751 | 0.803 | 0.851 | 0.906 | 0.96 | 1.015 | 1.072 | 1.157 | 1.246 |
|  |  | VS FA (incong) | 0.539 | 0.587 | 0.669 | 0.751 | 0.803 | 0.851 | 0.906 | 0.96 | 1.015 | 1.072 | 1.157 | 1.246 |
|  |  | VS FS (cong) | 0.532 | 0.623 | 0.691 | 0.762 | 0.826 | 0.893 | 0.946 | 0.995 | 1.053 | 1.11 | 1.16 | 1.212 |
|  |  | VA FS (incong) | 0.532 | 0.623 | 0.691 | 0.762 | 0.826 | 0.893 | 0.946 | 0.995 | 1.053 | 1.11 | 1.16 | 1.212 |
| molen | JW07 | VA FA (cong) | 0.534 | 0.617 | 0.681 | 0.745 | 0.78 | 0.816 | 0.878 | 0.942 | 1.003 | 1.067 | 1.133 | 1.206 |
|  |  | VS FA (incong) | 0.534 | 0.617 | 0.681 | 0.745 | 0.78 | 0.816 | 0.878 | 0.942 | 1.003 | 1.067 | 1.133 | 1.206 |
|  |  | VS FS (cong) | 0.529 | 0.62 | 0.666 | 0.713 | 0.744 | 0.774 | 0.836 | 0.901 | 0.971 | 1.041 | 1.108 | 1.175 |
|  |  | VA FS (incong) | 0.529 | 0.62 | 0.666 | 0.713 | 0.744 | 0.774 | 0.836 | 0.901 | 0.971 | 1.041 | 1.108 | 1.175 |
| molen | LN08 | VA FA (cong) | 0.525 | 0.654 | 0.693 | 0.735 | 0.823 | 0.914 | 0.97 | 1.025 | 1.082 | 1.138 | 1.242 | 1.341 |
|  |  | VS FA (incong) | 0.525 | 0.654 | 0.693 | 0.735 | 0.823 | 0.914 | 0.97 | 1.025 | 1.082 | 1.138 | 1.242 | 1.341 |
|  |  | VS FS (cong) | 0.52 | 0.586 | 0.612 | 0.639 | 0.678 | 0.722 | 0.769 | 0.819 | 0.873 | 0.923 | 1.015 | 1.108 |
|  |  | VA FS (incong) | 0.52 | 0.586 | 0.612 | 0.639 | 0.678 | 0.722 | 0.769 | 0.819 | 0.873 | 0.923 | 1.015 | 1.108 |
| molen | MB10 | VA FA (cong) | 0.552 | 0.615 | 0.652 | 0.686 | 0.722 | 0.758 | 0.804 | 0.851 | 0.898 | 0.948 | 1.02 | 1.098 |
|  |  | VS FA (incong) | 0.552 | 0.615 | 0.652 | 0.686 | 0.722 | 0.758 | 0.804 | 0.851 | 0.898 | 0.948 | 1.02 | 1.098 |
|  |  | VS FS (cong) | 0.525 | 0.59 | 0.643 | 0.702 | 0.73 | 0.755 | 0.798 | 0.846 | 0.894 | 0.943 | 1.021 | 1.096 |
|  |  | VA FS (incong) | 0.525 | 0.59 | 0.643 | 0.702 | 0.73 | 0.755 | 0.798 | 0.846 | 0.894 | 0.943 | 1.021 | 1.096 |
| molen | LG11 | VA FA (cong) | 0.542 | 0.605 | 0.65 | 0.696 | 0.743 | 0.791 | 0.841 | 0.892 | 0.94 | 0.991 | 1.067 | 1.139 |
|  |  | VS FA (incong) | 0.542 | 0.605 | 0.65 | 0.696 | 0.743 | 0.791 | 0.841 | 0.892 | 0.94 | 0.991 | 1.067 | 1.139 |
|  |  | VS FS (cong) | 0.536 | 0.628 | 0.681 | 0.735 | 0.789 | 0.846 | 0.901 | 0.957 | 1.013 | 1.067 | 1.136 | 1.214 |
|  |  | VA FS (incong) | 0.536 | 0.628 | 0.681 | 0.735 | 0.789 | 0.846 | 0.901 | 0.957 | 1.013 | 1.067 | 1.136 | 1.214 |
| molen | TL12 | VA FA (cong) | 0.516 | 0.563 | 0.598 | 0.634 | 0.66 | 0.686 | 0.729 | 0.773 | 0.823 | 0.871 | 0.928 | 0.985 |
|  |  | VS FA (incong) | 0.516 | 0.563 | 0.598 | 0.634 | 0.66 | 0.686 | 0.729 | 0.773 | 0.823 | 0.871 | 0.928 | 0.985 |
|  |  | VS FS (cong) | 0.523 | 0.621 | 0.66 | 0.699 | 0.731 | 0.763 | 0.808 | 0.857 | 0.901 | 0.949 | 1.034 | 1.126 |
|  |  | VA FS (incong) | 0.523 | 0.621 | 0.66 | 0.699 | 0.731 | 0.763 | 0.808 | 0.857 | 0.901 | 0.949 | 1.034 | 1.126 |
| *molen* |  | *Mean* | *0.532* | *0.610* | *0.663* | *0.717* | *0.764* | *0.812* | *0.864* | *0.918* | *0.974* | *1.03* | *1.112* | *1.194* |
| namil | WK01 | VA FA (cong) | 0.516 | 0.63 | 0.7 | 0.768 | 0.824 | 0.877 | 0.939 | 0.998 | 1.055 | 1.11 | 1.199 | 1.287 |
|  |  | VS FA (incong) | 0.516 | 0.63 | 0.7 | 0.768 | 0.824 | 0.877 | 0.939 | 0.998 | 1.055 | 1.11 | 1.199 | 1.287 |
|  |  | VS FS (cong) | 0.546 | 0.597 | 0.638 | 0.676 | 0.719 | 0.764 | 0.806 | 0.847 | 0.89 | 0.931 | 0.969 | 1.007 |
|  |  | VA FS (incong) | 0.546 | 0.597 | 0.638 | 0.676 | 0.719 | 0.764 | 0.806 | 0.847 | 0.89 | 0.931 | 0.969 | 1.007 |
| namil | HO05 | VA FA (cong) | 0.527 | 0.586 | 0.64 | 0.695 | 0.742 | 0.79 | 0.854 | 0.923 | 0.986 | 1.043 | 1.105 | 1.169 |
|  |  | VS FA (incong) | 0.527 | 0.586 | 0.64 | 0.695 | 0.742 | 0.79 | 0.854 | 0.923 | 0.986 | 1.043 | 1.105 | 1.169 |
|  |  | VS FS (cong) | 0.527 | 0.577 | 0.619 | 0.662 | 0.693 | 0.723 | 0.782 | 0.848 | 0.912 | 0.979 | 1.05 | 1.116 |
|  |  | VA FS (incong) | 0.527 | 0.577 | 0.619 | 0.662 | 0.693 | 0.723 | 0.782 | 0.848 | 0.912 | 0.979 | 1.05 | 1.116 |
| namil | MK06 | VA FA (cong) | 0.543 | 0.611 | 0.699 | 0.781 | 0.854 | 0.925 | 0.99 | 1.051 | 1.114 | 1.175 | 1.238 | 1.299 |
|  |  | VS FA (incong) | 0.543 | 0.611 | 0.699 | 0.781 | 0.854 | 0.925 | 0.99 | 1.051 | 1.114 | 1.175 | 1.238 | 1.299 |
|  |  | VS FS (cong) | 0.536 | 0.592 | 0.654 | 0.715 | 0.765 | 0.822 | 0.872 | 0.919 | 0.972 | 1.023 | 1.109 | 1.196 |
|  |  | VA FS (incong) | 0.536 | 0.592 | 0.654 | 0.715 | 0.765 | 0.822 | 0.872 | 0.919 | 0.972 | 1.023 | 1.109 | 1.196 |
| namil | JW07 | VA FA (cong) | 0.513 | 0.614 | 0.681 | 0.748 | 0.793 | 0.836 | 0.907 | 0.976 | 1.043 | 1.105 | 1.158 | 1.215 |
|  |  | VS FA (incong) | 0.513 | 0.614 | 0.681 | 0.748 | 0.793 | 0.836 | 0.907 | 0.976 | 1.043 | 1.105 | 1.158 | 1.215 |
|  |  | VS FS (cong) | 0.513 | 0.578 | 0.622 | 0.664 | 0.701 | 0.733 | 0.787 | 0.84 | 0.892 | 0.952 | 1 | 1.052 |
|  |  | VA FS (incong) | 0.513 | 0.578 | 0.622 | 0.664 | 0.701 | 0.733 | 0.787 | 0.84 | 0.892 | 0.952 | 1 | 1.052 |
| namil | LN08 | VA FA (cong) | 0.549 | 0.635 | 0.678 | 0.721 | 0.808 | 0.901 | 0.955 | 1.006 | 1.06 | 1.107 | 1.22 | 1.347 |
|  |  | VS FA (incong) | 0.549 | 0.635 | 0.678 | 0.721 | 0.808 | 0.901 | 0.955 | 1.006 | 1.06 | 1.107 | 1.22 | 1.347 |
|  |  | VS FS (cong) | 0.529 | 0.633 | 0.67 | 0.708 | 0.744 | 0.782 | 0.832 | 0.888 | 0.941 | 1.003 | 1.074 | 1.148 |
|  |  | VA FS (incong) | 0.529 | 0.633 | 0.67 | 0.708 | 0.744 | 0.782 | 0.832 | 0.888 | 0.941 | 1.003 | 1.074 | 1.148 |
| namil | MB10 | VA FA (cong) | 0.536 | 0.575 | 0.617 | 0.657 | 0.704 | 0.753 | 0.793 | 0.827 | 0.865 | 0.907 | 0.992 | 1.072 |
|  |  | VS FA (incong) | 0.536 | 0.575 | 0.617 | 0.657 | 0.704 | 0.753 | 0.793 | 0.827 | 0.865 | 0.907 | 0.992 | 1.072 |
|  |  | VS FS (cong) | 0.515 | 0.614 | 0.652 | 0.689 | 0.739 | 0.787 | 0.827 | 0.865 | 0.901 | 0.943 | 1.012 | 1.083 |
|  |  | VA FS (incong) | 0.515 | 0.614 | 0.652 | 0.689 | 0.739 | 0.787 | 0.827 | 0.865 | 0.901 | 0.943 | 1.012 | 1.083 |
| namil | LG11 | VA FA (cong) | 0.539 | 0.596 | 0.642 | 0.684 | 0.733 | 0.79 | 0.861 | 0.93 | 0.993 | 1.053 | 1.108 | 1.163 |
|  |  | VS FA (incong) | 0.539 | 0.596 | 0.642 | 0.684 | 0.733 | 0.79 | 0.861 | 0.93 | 0.993 | 1.053 | 1.108 | 1.163 |
|  |  | VS FS (cong) | 0.546 | 0.582 | 0.639 | 0.693 | 0.772 | 0.855 | 0.909 | 0.97 | 1.026 | 1.086 | 1.142 | 1.199 |
|  |  | VA FS (incong) | 0.546 | 0.582 | 0.639 | 0.693 | 0.772 | 0.855 | 0.909 | 0.97 | 1.026 | 1.086 | 1.142 | 1.199 |
| namil | TL12 | VA FA (cong) | 0.518 | 0.536 | 0.562 | 0.587 | 0.626 | 0.669 | 0.718 | 0.763 | 0.813 | 0.864 | 0.925 | 0.988 |
|  |  | VS FA (incong) | 0.518 | 0.536 | 0.562 | 0.587 | 0.626 | 0.669 | 0.718 | 0.763 | 0.813 | 0.864 | 0.925 | 0.988 |
|  |  | VS FS (cong) | 0.513 | 0.679 | 0.724 | 0.768 | 0.803 | 0.839 | 0.892 | 0.937 | 0.986 | 1.037 | 1.108 | 1.184 |
|  |  | VA FS (incong) | 0.513 | 0.679 | 0.724 | 0.768 | 0.803 | 0.839 | 0.892 | 0.937 | 0.986 | 1.037 | 1.108 | 1.184 |
| *namil* |  | *Mean* | *0.529* | *0.602* | *0.652* | *0.701* | *0.751* | *0.803* | *0.858* | *0.912* | *0.966* | *1.020* | *1.088* | *1.158* |
| loman | WK01 | VA FA (cong) | 0.542 | 0.714 | 0.814 | 0.922 | 0.98 | 1.04 | 1.128 | 1.216 | 1.291 | 1.375 | 1.417 | 1.455 |
|  |  | VS FA (incong) | 0.542 | 0.714 | 0.814 | 0.922 | 0.98 | 1.04 | 1.128 | 1.216 | 1.291 | 1.375 | 1.417 | 1.455 |
|  |  | VS FS (cong) | 0.545 | 0.577 | 0.616 | 0.656 | 0.697 | 0.738 | 0.782 | 0.831 | 0.88 | 0.93 | 0.993 | 1.055 |
|  |  | VA FS (incong) | 0.545 | 0.577 | 0.616 | 0.656 | 0.697 | 0.738 | 0.782 | 0.831 | 0.88 | 0.93 | 0.993 | 1.055 |
| loman | HO05 | VA FA (cong) | 0.545 | 0.578 | 0.627 | 0.677 | 0.735 | 0.793 | 0.851 | 0.911 | 0.97 | 1.032 | 1.104 | 1.183 |
|  |  | VS FA (incong) | 0.545 | 0.578 | 0.627 | 0.677 | 0.735 | 0.793 | 0.851 | 0.911 | 0.97 | 1.032 | 1.104 | 1.183 |
|  |  | VS FS (cong) | 0.514 | 0.552 | 0.588 | 0.627 | 0.669 | 0.708 | 0.768 | 0.829 | 0.89 | 0.952 | 1.034 | 1.113 |
|  |  | VA FS (incong) | 0.514 | 0.552 | 0.588 | 0.627 | 0.669 | 0.708 | 0.768 | 0.829 | 0.89 | 0.952 | 1.034 | 1.113 |
| loman | MK06 | VA FA (cong) | 0.529 | 0.617 | 0.715 | 0.816 | 0.864 | 0.913 | 0.965 | 1.02 | 1.073 | 1.131 | 1.194 | 1.261 |
|  |  | VS FA (incong) | 0.529 | 0.617 | 0.715 | 0.816 | 0.864 | 0.913 | 0.965 | 1.02 | 1.073 | 1.131 | 1.194 | 1.261 |
|  |  | VS FS (cong) | 0.517 | 0.577 | 0.648 | 0.719 | 0.77 | 0.824 | 0.884 | 0.949 | 1.01 | 1.081 | 1.132 | 1.185 |
|  |  | VA FS (incong) | 0.517 | 0.577 | 0.648 | 0.719 | 0.77 | 0.824 | 0.884 | 0.949 | 1.01 | 1.081 | 1.132 | 1.185 |
| loman | JW07 | VA FA (cong) | 0.515 | 0.6 | 0.65 | 0.705 | 0.761 | 0.817 | 0.888 | 0.965 | 1.038 | 1.112 | 1.173 | 1.24 |
|  |  | VS FA (incong) | 0.515 | 0.6 | 0.65 | 0.705 | 0.761 | 0.817 | 0.888 | 0.965 | 1.038 | 1.112 | 1.173 | 1.24 |
|  |  | VS FS (cong) | 0.52 | 0.586 | 0.621 | 0.651 | 0.697 | 0.747 | 0.815 | 0.883 | 0.947 | 1.013 | 1.059 | 1.101 |
|  |  | VA FS (incong) | 0.52 | 0.586 | 0.621 | 0.651 | 0.697 | 0.747 | 0.815 | 0.883 | 0.947 | 1.013 | 1.059 | 1.101 |
| loman | LN08 | VA FA (cong) | 0.515 | 0.623 | 0.664 | 0.703 | 0.767 | 0.829 | 0.898 | 0.964 | 1.029 | 1.101 | 1.21 | 1.327 |
|  |  | VS FA (incong) | 0.515 | 0.623 | 0.664 | 0.703 | 0.767 | 0.829 | 0.898 | 0.964 | 1.029 | 1.101 | 1.21 | 1.327 |
|  |  | VS FS (cong) | 0.516 | 0.609 | 0.638 | 0.667 | 0.695 | 0.717 | 0.779 | 0.846 | 0.911 | 0.98 | 1.083 | 1.189 |
|  |  | VA FS (incong) | 0.516 | 0.609 | 0.638 | 0.667 | 0.695 | 0.717 | 0.779 | 0.846 | 0.911 | 0.98 | 1.083 | 1.189 |
| loman | MB10 | VA FA (cong) | 0.534 | 0.577 | 0.603 | 0.629 | 0.673 | 0.716 | 0.766 | 0.813 | 0.864 | 0.916 | 0.982 | 1.052 |
|  |  | VS FA (incong) | 0.534 | 0.577 | 0.603 | 0.629 | 0.673 | 0.716 | 0.766 | 0.813 | 0.864 | 0.916 | 0.982 | 1.052 |
|  |  | VS FS (cong) | 0.502 | 0.583 | 0.617 | 0.654 | 0.69 | 0.728 | 0.773 | 0.818 | 0.869 | 0.923 | 1.007 | 1.091 |
|  |  | VA FS (incong) | 0.502 | 0.583 | 0.617 | 0.654 | 0.69 | 0.728 | 0.773 | 0.818 | 0.869 | 0.923 | 1.007 | 1.091 |
| loman | LG11 | VA FA (cong) | 0.528 | 0.646 | 0.691 | 0.732 | 0.79 | 0.856 | 0.918 | 0.984 | 1.049 | 1.115 | 1.189 | 1.263 |
|  |  | VS FA (incong) | 0.528 | 0.646 | 0.691 | 0.732 | 0.79 | 0.856 | 0.918 | 0.984 | 1.049 | 1.115 | 1.189 | 1.263 |
|  |  | VS FS (cong) | 0.53 | 0.604 | 0.66 | 0.716 | 0.77 | 0.826 | 0.894 | 0.964 | 1.036 | 1.112 | 1.192 | 1.273 |
|  |  | VA FS (incong) | 0.53 | 0.604 | 0.66 | 0.716 | 0.77 | 0.826 | 0.894 | 0.964 | 1.036 | 1.112 | 1.192 | 1.273 |
| loman | TL12 | VA FA (cong) | 0.511 | 0.541 | 0.561 | 0.583 | 0.614 | 0.65 | 0.704 | 0.763 | 0.817 | 0.876 | 0.921 | 0.965 |
|  |  | VS FA (incong) | 0.511 | 0.541 | 0.561 | 0.583 | 0.614 | 0.65 | 0.704 | 0.763 | 0.817 | 0.876 | 0.921 | 0.965 |
|  |  | VS FS (cong) | 0.512 | 0.624 | 0.652 | 0.678 | 0.712 | 0.752 | 0.798 | 0.848 | 0.899 | 0.951 | 1.028 | 1.109 |
|  |  | VA FS (incong) | 0.512 | 0.624 | 0.652 | 0.678 | 0.712 | 0.752 | 0.798 | 0.848 | 0.899 | 0.951 | 1.028 | 1.109 |
| *loman* |  | *Mean* | *0.523* | *0.601* | *0.648* | *0.696* | *0.743* | *0.791* | *0.851* | *0.93* | *0.973* | *1.038* | *1.107* | *1.179* |

Note: VA FA (cong) – “vocal anger, facial anger (congruent)”, VS FA (incong) – “vocal surprise, facial anger (incongruent)”, VS FS (cong) – “vocal surprise, facial surprise (congruent)”, VA FS (incong) – “vocal anger, facial surprise (incongruent)”, all values in seconds

#

# **5 Reference**

Schaefer, A., Nils, F., Sanchez, X., & Philippot, P. (2010). Assessing the effectiveness of a large database of emotion-eliciting films: A new tool for emotion researchers. *Cognition & Emotion, 24*(7), 1153–1172. https://doi.org/10.1080/02699930903274322
